# Supplementary material for: Gender Differences in the Impact of COVID-19 Pandemic on Mental Health of Italian Academic Workers
Source: J Pers Med. 2022 Apr 11;12(4):613. doi: 10.3390/jpm12040613 (PMC9030948; doi:10.3390/jpm12040613)
Supplement: Supplementary file 1 [file jpm-12-00613-s001.zip › jpm-1661599-supplementary.pdf]

Survey on Covid-19

UNISA "Comitato Unico di Garanzia"

1) **Type of employment at Unisa**
    A. Professors/ Researchers
    B. Administrative officers/ Technicians
    C. Others (students, post-doctoral fellows or visiting fellows)
2) **Age**
    A. 20-40 years old
    B. 40-60 years old
    C. > 60 years old
3) **Gender**
    A. Male
    B. Female
    C. Other
4) **Number of family member**
    A. 1 person
    B. 2 people
    C. 3 people
    D. More than 3 people
5) **Children' age? (More than one answer possible)**
    A. 0-3 years old
    B. 4-6 years old
    C. 7-13 years old
    D. > 18 years old
    E. No children
6) **Are you a caregiver for a family member with disability? (More than one answer possible)**
    A. No
    B. Yes, my partner
    C. Yes, my child
    D. Yes, other family member living with me
    E. Yes, other family member not living with me
7) **Which of the following might help in balancing work and private life? 7.1 Possibility to choose between teleworking and smart working. 7.2 Possibility to choose hours and days of teleworking. 7.3 Availability of childcare services.**
    A. Relevant
    B. Partially relevant
    C. No influence
    D. Mainly irrelevant
    E. Irrelevant
8) **During this period, have you teleworked?**
    A. No
    B. Yes, every working day
    C. Yes, partially

9) Do you have any electronic device at home (personal computer, notebook, tablet…)?

A. No
B. Yes

If yes, how many.

10) Are your electronic devices enough for your family need?

A. Completely adequate
B. Just enough
C. No

11) Is your internet connection adequate?

A. Completely adequate
B. Just enough
C. No

12) Has your work performance changed during teleworking?

A. No
B. Yes
C. Yes, only at the beginning

13) Your home-to-workplace distance

A. < 50 km
B. > 50 km
C. > 100 km
D. Same town/city

14) Might teleworking reduce daily expenses to reach the workplace?

A. No
B. Yes
C. No influence

15) Might teleworking help balancing between private life and work?

A. No
B. Yes
C. No influence

16) How do you rate your experience with teleworking?

A. Very positive
B. Positive
C. Negative
D. Very negative

17) Are you willing to telework in the future?

A. Yes, all working days
B. Yes, only for few days per week
C. Yes, only if necessary
D. No, it is not productive
E. No, my job requires to stay on place

18) Please describe how to improve teleworking experience.

Free answer.

19) How much are you worried about Covid-19?

A. Extremely worried
B. Moderately worried
C. No worried

21) **Which of the following activities have you practiced more during pandemic? (More than one answer possible)**
   A. Physical exercise at home
   B. Research / work / study
   C. Cooking
   D. Activities with family members
   E. Reading/ watching tv
   F. Home repairs
   G. Relax

22) **Which of the following moods do you have in this period? (More than one answer possible)**
   A. Resignation
   B. Insomnia
   C. Loneliness
   D. Sadness
   E. Exasperation
   F. Fear
   G. Insecurity
   H. Anxiety
   I. Concern
   J. Tranquility
   K. Ease
   L. Pleasure

23) **How did you manage relationships with not cohabitant people?**
   A. I increased phone calls with them
   B. I took care of them going to their homeplace
   C. I dramatically reduced my relationships with others
   D. Other (specify)

24) **What did you do to reduce Covid-19 risk of infection? (More than one answer possible)**
   A. Go outside only if necessary
   B. Stay at home
   C. Wear a mask/wash hands/physical distancing
   D. Surface sanitation

25) **Is your home area enough for you?**
   A. Completely adequate
   B. Just enough
   C. No

26) **After stay-at-home shelter, how your relationships with cohabitants have changed? (More than one answer possible)**
   A. We increased activities together
   B. We intensified our conversations
   C. We have more stress
   D. We have less privacy
   E. No modifications

27) **Which of the following moods are prevalent in your cohabitants? (More than one answer possible)**
   A. I am living alone
   B. Stress
   C. Availability
   D. Tranquility
   E. Restlessness
   F. Anger
   G. Discouragement

28) **During stay-at-home shelter, have you suffered or forced someone?**
   A. No
   B. Yes

29) **During stay-at-home shelter, have you witnessed any form of harassment?**
   A. No
   B. Yes

30) **During stay-at-home shelter, have you been harassed, or have you forced someone?**
   A. No
   B. Yes

31) **What do you think could have been avoided during stay-at-home shelter?**
   Free answer.

32) **Do you know the 1522 number for domestic violence support and for stalking support?**
   A. No
   B. Yes
